# Genetic analysis of haplotype data for 23 Y-chromosome short tandem repeat loci in the Turkish population recently settled in Sarajevo, Bosnia and Herzegovina

**DOI:** 10.3325/cmj.2014.55.530

**Published:** 2014-10

**Authors:** Serkan Dogan, Dragan Primorac, Damir Marjanović

**Affiliations:** 1 International Burch University, Department of Genetics and Bioengineering, Sarajevo, Bosnia and Herzegovina; 2 University of Split School of Medicine and Department of Forensic Science, Split, Croatia; 3 University of Osijek School of Medicine, Osijek, Croatia; 4 Eberly College of Science, Penn State University, University Park, PA, USA; 5 University of New Haven, New Haven, CT, USA; 6 University of Sarajevo, Institute for Genetic Engineering and Biotechnology, Sarajevo, Bosnia and Herzegovina

## Abstract

**Aim:**

To explore the distribution and polymorphisms of 23 short tandem repeat (STR) loci on the Y chromosome in the Turkish population recently settled in Sarajevo, Bosnia and Herzegovina and to investigate its genetic relationships with the homeland Turkish population and neighboring populations.

**Methods:**

This study included 100 healthy unrelated male individuals from the Turkish population living in Sarajevo. Buccal swab samples were collected as a DNA source. Genomic DNA was extracted using the salting out method and amplification was performed using PowerPlex Y 23 amplification kit. The studied population was compared to other populations using pairwise genetic distances, which were represented with a multi-dimensional scaling plot.

**Results:**

Haplotype and allele frequencies of the sample population were calculated and the results showed that all 100 samples had unique haplotypes. The most polymorphic locus was DYS458, and the least polymorphic DYS391. The observed haplotype diversity was 1.0000 ± 0.0014, with a discrimination capacity of 1.00 and the match probability of 0.01. *R_st_* values showed that our sample population was closely related in both dimensions to the Lebanese and Iraqi populations, while it was more distant from Bosnian, Croatian, and Macedonian populations.

**Conclusion:**

Turkish population residing in Sarajevo could be observed as a representative Turkish population, since our results were consistent with those previously published for the homeland Turkish population. Also, this study once again proved that geographically close populations were genetically more related to each other.

Human Y chromosome short tandem repeats (Y-STRs) are repeating regions with 2-7 bp long repetitive units found in the non-recombining region of Y chromosome. Y-STRs are characterized by male inheritance pattern. They are the most widely used Y chromosome markers due to simple typing and a high level of diversity. Typing is performed using polymerase chain reaction (PCR), which is a reliable procedure tolerant to degraded DNA. Thus, Y-STRs can be used in forensics for the investigation of sexual assault cases, for deficient paternity testing when the alleged father is not available for testing, in gang rape situations (mixture of two or more male DNA samples), for the investigation of genetic reasons of male infertility, in genealogical research, particularly for surname testing, in population genetic studies, for the verification of amelogenin Y-deficient men, and in genetic epidemiology (1-9). In this study genotyping was performed by PowerPlex Y 23 kit (Promega Corporation, Madison, WI, USA). This kit types 23 Y-STR loci in a tested haplotype and includes 6 new Y-STR loci when compared to the previous Y-STR commercial kits, namely DYS576, DYS481, DYS549, DYS533, DYS570, and DYS643 (10,11).

More than 400 years of shared history of Bosnia and Herzegovina and Ottoman Empire shaped cultural features of both populations, with consequences visible even in the modern era. However, according to a previously published study (12), there was no greater genetic impact on the local population. Nowadays, for the first time we have a considerable settlement process from Turkey to Bosnia and Herzegovina, which could have a certain impact on local population diversity.

This study included Turkish students currently studying in Sarajevo as a representative sample of the Turkish population that has recently settled in Sarajevo. The aim of the study was to provide the haplotype polymorphisms and distributions in the Turkish population and estimate their forensic parameters. In addition, population pairwise genetic distances (*R_st_*) and associated probability values (*P* values) with 10 000 permutations were calculated between the studied population and the neighboring populations. Multi-dimensional scaling (MDS) plots were generated using genetic distances for the comparison of different populations’ haplotype data found in the Y Chromosome Haplotype Reference Database (YHRD, *www.yhrd.org*).

## Materials and methods

### Study population

A total of 100 unrelated (not belonging to the same nuclear family) healthy male students from Turkish population living in Sarajevo and originating from different geographical regions of Turkey were sampled for the Y-STR analysis. DNA samples were collected at the International Burch University in the period between March, 2013 and January, 2014. The informed consent form was obtained from all participants. Ethical approval was received from the International Burch University, Sarajevo.

### Sample preparation and DNA extraction

Buccal swabs were collected and used as a DNA source (n = 100). Air-dried buccal swabs were placed in paper envelopes and stored at +4°C for DNA extraction. Genomic DNA was isolated from buccal swabs using the salting out method (13) and extracted DNA samples were stored at -20°C until PCR amplification.

### STR amplification and genotyping

PCR amplification of 23 STR loci (DYS19, DYS385a/b, DYS389I/II, DYS390, DYS391, DYS392, DYS393, DYS437, DYS438, DYS439, DYS448, DYS456, DYS458, DYS481, DYS533, DYS549, DYS570, DYS576, DYS635, DYS643, and GATA H4) on Y chromosome was performed using PowerPlex Y 23 System (Promega Corporation) according to the manufacturer’s recommendations (14). PCR was performed using GeneAmp PCR System thermal cycler (Applied Biosystems, Foster City, CA, USA) according to the manufacturer’s recommendations. PowerPlex Y 23 PCR amplicons were analyzed on ABI PRISM 310 Genetic Analyzer (Applied Biosystems). Collected data were analyzed and haplotypes were obtained using GeneMapper software version 3.2 (Applied Biosystems). Determination of amplified allele designations was performed by comparing the amplicons to those in allelic ladders of the PowerPlex Y 23 kit.

### Statistical analyses

Allele and haplotype frequencies were calculated by counting method. Haplotype diversity was estimated by Nei’s formula (15): HD = (1- ∑ *p_i_^2^*)*n/(n-1), where n is the sample size and p_i_ is the *i*th’s haplotype frequency. Gene diversity (GD) was calculated as 1- ∑ *p_i_^2^*, where *p_i_* is the allele frequency. Arlequin software version 3.5 was used (16). The match probability (MP) was calculated as ∑ *p_i_^2^*, where *p_i_* is the frequency of the *i*th haplotype. The discrimination capacity was calculated according to the formula DC = h/n, where h is the number of different haplotypes in the observed population (17). In order to compare the countries located in the proximity to Bosnia and Herzegovina and Turkey, we used the Y Chromosome Haplotype Reference Database – YHRD (*www.yhrd.org*) (18). AMOVA online tool (19) from YHRD was used to calculate population pairwise genetic distances (*R_st_*) and associated probability values (*P values*) between the studied population and the neighboring populations with 10 000 permutations. MDS plots were generated using genetic distances for the comparison of different populations’ haplotype data present in YHRD. For the population comparison, recently published haplotype data present in YHRD were used and the analyses were performed on 23 Y-chromosome STR loci. Compared population samples with the number of haplotypes were as follows: Macedonian (n = 101), Italian (n = 335), Lebanese (n = 555), Iraqi (n = 124), Greek (n = 109), Slovenian (n = 104), Hungarian (n = 143), German (n = 131), Croatian (n = 125), Bosnian (n = 100), and Turkish (n = 100).

## Results

We calculated haplotype frequencies of the sample population and detected 165 alleles at the 23 Y-STR loci (Table 1). All 100 samples had unique Y 23 haplotypes, which is extremely satisfying and demonstrates the power of the new PowerPlex Y 23 kit (20). Apart from DYS385a/b, the most polymorphic locus was DYS458 with 12 alleles. High degree of polymorphism was also observed in all the six loci that were analyzed for the first time using PowerPlex Y 23 kit. The least polymorphic loci were DYS391 with 3 alleles, DYS438 with 4 alleles, and DYS389I, DYS437, DYS439, and DYS393 with 5 alleles. Haplotype diversity was 1.0000 ± 0.0014 with a DC of 1.00 and MP of 0.01.

**Table 1:** Allele frequencies and gene diversity values for the 23 Y-short tandem repeat loci in the Turkish population living in Sarajevo, Bosnia and Herzegovina*^†^

**DYS576** GD=0.799		**DYS389I** GD=0.639		**DYS448** GD=0.718		**DYS389II** GD=0.757		**DYS19** GD=0.679		**DYS391** GD=0.470	
**Allele**	Frequency	Allele	Frequency	Allele	Frequency	Allele	Frequency	Allele	Frequency	Allele	Frequency
**14**	0.01	11	0.02	18	0.06	27	0.03	12	0.01	9	0.07
**15**	0.12	12	0.24	19	0.31	28	0.13	13	0.08	**10**	**0.68**
**16**	0.22	**13**	**0.50**	**20**	**0.38**	**29**	**0.35**	**14**	**0.40**	11	0.25
**17**	0.23	14	0.23	21	0.19	30	0.22	15	0.38		
**18**	**0.26**	15	0.01	22	0.04	31	0.23	15,16	0.02		
**19**	0.13			23	0.01	32	0.04	16	0.10		
**20**	0.03			24	0.01			17	0.01		
**DYS481** GD=0.799		**DYS549** GD=0.631		**DYS533** GD=0.652		**DYS438** GD=0.702		**DYS437** GD=0.653		**DYS570** GD=0.804	
**Allele**	Frequency	Allele	Frequency	Allele	Frequency	Allele	Frequency	Allele	Frequency	Allele	Frequency
**19**	0.01	10	0.02	9	0.03	9	0.32	12	0.01	14	0.01
**21**	0.15	11	0.16	10	0.12	**10**	**0.39**	13	0.02	15	0.08
**22**	**0.33**	**12**	**0.54**	11	0.36	11	0.17	**14**	**0.47**	16	0.17
**23**	0.21	13	0.22	**12**	**0.45**	12	0.12	15	0.26	**17**	**0.30**
**24**	0.11	14	0.05	13	0.03			16	0.24	18	0.23
**25**	0.09	15	0.01	14	0.01					19	0.12
**26**	0.07									20	0.05
**27**	0.02									21	0.02
**31**	0.01									22	0.02
**DYS570** GD=0.804		**DYS635** GD=0.762		**DYS390** GD=0.715		**DYS439** GD=0.707		**DYS392** GD=0.552		**DYS385a/b** GD=0.942	
**Allele**	Frequency	Allele	Frequency	Allele	Frequency	Allele	Frequency	Allele	Frequency	Alleles	Frequency
**14**	0.01	19	0.01	21	0.04	10	0.16	10	0.04	11,13	0.02
**15**	0.08	20	0.07	22	0.17	**11**	**0.41**	**11**	**0.64**	11,14	0.12
**16**	0.17	**21**	**0.40**	**23**	**0.44**	12	0.29	12	0.07	11,15	0.03
**17**	**0.30**	22	0.14	24	0.21	13	0.12	13	0.17	11,17	0.01
**18**	0.23	23	0.16	25	0.13	14	0.02	14	0.05	12,13	0.04
**19**	0.12	24	0.16	26	0.01			15	0.03	12,14	0.06
**20**	0.05	25	0.04							12,15	0.04
**21**	0.02	26	0.01							12,16	0.05
**22**	0.02	27	0.01							12,17	0.02
										12,19	0.02
										13,13	0.02
**DYS643**		**DYS393**		**DYS458**		**DYS456**		**GATA H4**		13,14	0.08
GD**=**0.733		GD=0.638		GD=0.813		GD=0.615		GD=0.700		13,15	0.07
**Allele**	Frequency	Allele	Frequency	Allele	Frequency	Allele	Frequency	Allele	Frequency	**13,16**	**0.11**
**7**	0.01	11	0.02	11	0.01	13	0.07	9	0.01	13,17	0.01
**8**	0.03	**12**	**0.46**	13	0.03	14	0.07	10	0.10	13,18	0.02
**9**	0.13	13	0.36	14	0.04	**15**	**0.58**	**11**	**0.41**	13,20	0.02
**10**	**0.41**	14	0.14	15	0.18	16	0.18	12	0.31	14,14	0.06
**11**	0.18	15	0.02	**16**	**0.30**	17	0.08	13	0.16	14,15	0.03
**12**	0.22			17	0.22	18	0.02	14	0.01	14,16	0.02
**13**	0.02			17.2	0.01					14,17	0.02
				18	0.10					14,18	0.01
				18.2	0.05					15,15	0.01
				19	0.03					15,16	0.03
				20	0.02					15,17	0.02
				22	0.01					15,19	0.01
										16,18	0.02
										16,20	0.01
										17,18	0.02

*The table shows allele frequencies for each investigated locus except for DYS385a/b, for which genotype frequencies were calculated for the combination of the two alleles. GD – gene diversity.

†Major allele frequencies per locus are in bold.

Average GD for the study population was 0.703, ranging from 0.470 to 0.942. The initial analysis of GD values indicated that the highest GD was detected at DYS385a/b loci with a value of 0.942 and the lowest GD at DYS391 locus with a value of 0.470, which is consistent with the polymorphism findings presented above in this study.

Two microvariant alleles were detected at DYS458, namely 17.2 and 18.2. The total frequency of the variant alleles at DYS458 was 6% of all study samples. We detected duplicated allele (alleles 15, 16) in the two samples at DYS19.

The choice of world populations for comparison with our data was mostly limited by the fact that the majority of populations still lack relevant data on 23 Y-STR loci. Turkish population was closer to the Lebanese (R*_st_* = 0.001, *P* = 0.289) and Iraqi (R*_st_* = -0.001, *P* = 0.522) populations than to Italian (R*_st_* = 0.027, *P* = 0.000) and Greek (R*_st_* = 0.039, *P* = 0.000) populations. Also, it was closer to Hungarian (R*_st_* = 0.074, *P* = 0.000), German (R*_st_* = 0.088, *P* = 0.000), and Slovenian (R*_st_* = 0.089, *P* = 0.000) populations than to Macedonian (R*_st_* = 0.126, *P* = 0.000) and Croatian (R*_st_* = 0.216, *P* = 0.000) populations. The greatest genetic distance was detected between Turkish and Bosnian (R*_st_* = 0.284, *P* = 0.000) population (Table 2).

**Table 2:** Analysis of molecular variance pairwise distances based on *R_st_* values between Turkish population from the present study and selected populations

Population*	LEB	GER	BBH	CRO	GRE	HUN	IR	SLO	MAC	ITA	TBH
[Lebanese] Lebanon (LEB)	-	0.0000	0.0000	0.0000	0.0000	0.0000	0.0925	0.0000	0.0000	0.0000	0.2888
[German] Germany (GER)	0.0859	-	0.0000	0.0000	0.0033	0.0729	0.0000	0.0078	0.0000	0.0000	0.0000
[Bosnian] Bosnia and Herzegovina (BBH)	0.2868	0.1714	-	0.1103	0.0000	0.0000	0.0000	0.0001	0.0000	0.0000	0.0000
[Croatian] Croatia (CRO)	0.2288	0.1055	0.0093	-	0.0000	0.0000	0.0000	0.0019	0.0000	0.0000	0.0000
[Greek] Greece (GRE)	0.0332	0.0233	0.1453	0.0963	-	0.0772	0.0028	0.0785	0.0036	0.0000	0.0001
[Hungarian] Hungary (HUN)	0.0782	0.0072	0.1253	0.0742	0.0084	-	0.0000	0.5782	0.0000	0.0000	0.0000
[Iraqi] Iraq (IR)	0.0041	0.0696	0.2708	0.1997	0.0237	0.0568	-	0.0001	0.0000	0.0000	0.5220
[Slovenian] Slovenia (SLO)	0.0905	0.0210	0.0942	0.0507	0.0102	-0.0025	0.0709	-	0.0004	0.0000	0.0000
[Macedonian] Macedonia (MAC)	0.1067	0.0903	0.1503	0.1251	0.0331	0.0602	0.1039	0.0620	-	0.0000	0.0000
[Italian] Italy (ITA)	0.0350	0.0676	0.3224	0.2490	0.0512	0.0767	0.0290	0.1080	0.1330	-	0.0000
[Turkish] Bosnia and Herzegovina (TBH)	0.0012	0.0877	0.2841	0.2164	0.0389	0.0740	0.0000	0.0891	0.1258	0.0272	-

*P values are shown above the diagonal and *R_st_* values below it. Population names in brackets refer to YHRD designations (*www.yhrd.org*).

**Figure 1:**
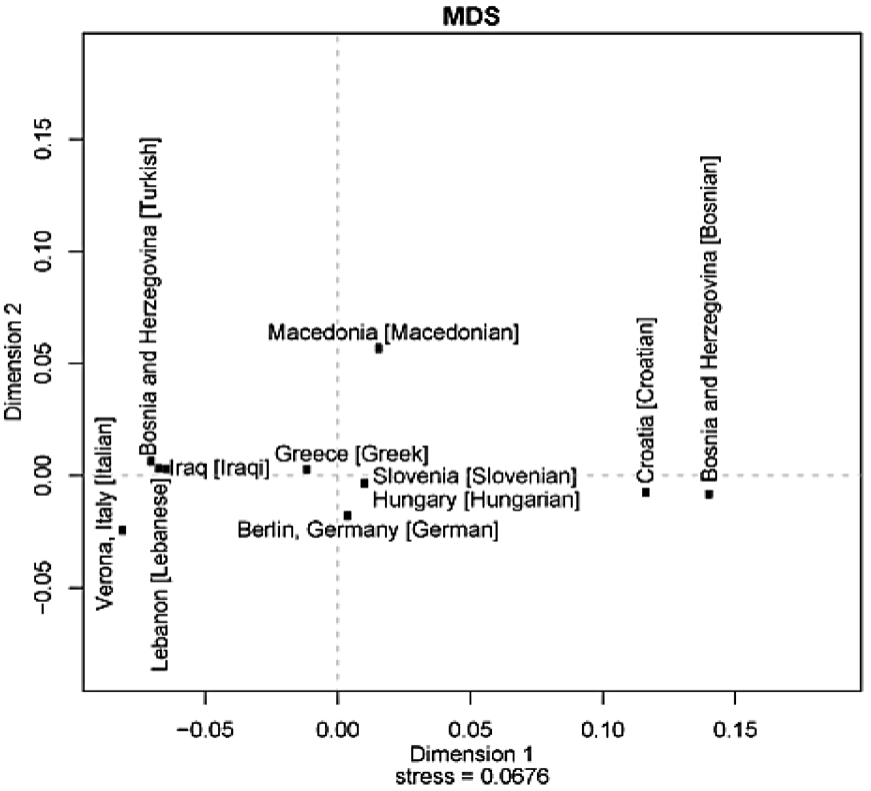
Multi-dimensional scaling (MDS) plot based on population pairwise *R_st_*values between compared populations

MDS plot was generated using pairwise *R_st_* values to estimate the genetic relationships between the compared populations (Figure 1). Our sample population was closely related to the Lebanese and Iraqi populations in both dimensions, while it was much more distant to Bosnian, Croatian and Macedonian populations.

## Discussion

A comparison of the Turkish population currently living in Sarajevo with other populations showed that populations that were geographically closer were also more similar to each other. Thus, the populations most similar to our population were Iraqi and Lebanese. On the other hand, European populations are geographically more distant from Turkey and they showed greater differences from our population. The most distant populations were those from the Balkan Peninsula: Bosnian, Croatian, and Macedonian. Another study has also found important differences between Turkish population and European populations (1). However, there are no studies comparing Turkish population with a greater number of other world populations, especially when it comes to the comparison over 23 Y-STRs.

In the recent years, Y-STR marker analysis has been increasingly used in forensic science and population studies. Studies including 7, 9, 11, or 17 Y-STRs have been made for Turkish population. However, this study gives the first population data for 23 Y-STR loci for Turkish population in Eurasia region, which is one of the biggest advantages of the study as it will surely improve the knowledge about the genetic characteristics of the Turkish population. YHRD database is missing data on 23 Y-STR loci for Turkish population and this study can be helpful in filling that gap in the literature. Although sampled in Sarajevo, the population from the present study could be considered representative of the Turkish population in general since our data are similar to those obtained in 2003 (21). It is worth noting that the Turkish population sampled for this research comes from different parts of Turkey and can represent Turkish population on a small scale.

Furthermore, one of our intentions was also to “genetically record” a preliminary situation within this Turkish population, as well as the relationships with the local and neighboring populations, but also with the homeland Turkish population. These data can be used as a starting point for future population studies of Turkish population in the region, which currently shows a tendency to expand.

Also, in the examined population, high degree of haplotype diversity was observed as all haplotypes were unique (no profile appeared more than once), which is not usually observed in the literature and confirms that the tested individuals are indeed paternally unrelated. Such results are probably a consequence of using PowerPlex Y 23 kit, which types results on 23 Y-STR loci, thus enabling increased haplotype diversity to be observed. A large number of identified alleles also confirm high haplotype diversity due to the use of 23 Y-STR loci, while previous studies of Turkish population showed a much smaller number of alleles due to the use of fewer Y-STR loci (1,21-24).

Six loci that were included and analyzed for the first time in PowerPlex Y 23 kit (DYS576, DYS481, DYS549, DYS533, DYS570, and DYS643) were found to be highly informative and to contribute to the uniqueness of the haplotypes in the present population. DYS385a/b loci showed a high degree of polymorphism, which was expected as these loci were analyzed as a duplicated locus in the generated haplotypes. It was also found that DYS458 was one of the most informative markers with 12 different alleles. This marker was shown to be highly polymorphic in a study of Turkish population from Anatolia as well (22).

The least informative locus was DYS391, with GD value of 0.470 and with only 3 alleles, which is in concordance with the previously published data for Turkish population (1), as well as with older studies (21,23,24) that used a smaller number of Y-STR loci. One of these studies (21) typed 6 Y-STR loci but the observed results were similar. One of the least informative loci in our study was DYS437 with 5 alleles, which confirmed the results of a previous study of Anatolian population (22), in which this locus was the least informative with 3 alleles.

Two of 12 alleles on locus DYS458 were microvariant alleles (17.2 and 18.2). The appearance of variant alleles was expected as it is a specific feature of the locus DYS458 (6). These alleles are most frequently found in the Northern and the Eastern Africa and the Caucasus but are less common in Europe. Having such variant alleles detected in a population increases the information content of the haplotypes, which is why we consider it an especially important finding.

Duplication was found in two individuals at DYS19 locus, which was also expected according to the theoretic and experimental data from the literature (2). Both individuals had the same genotype: 15, 16. Identical situation was observed in a study of Turkish population from the Central Anatolia (24). In this study, one individual had duplication at DYS19 locus with the combination 15, 16.

To conclude, 23 Y-STR loci had high haplotype diversity and the kits using this number of Y-STR loci represent great progression in the field of Y chromosome-related testing of individuals for the purposes of forensic investigation, paternity testing, population studies, and genealogical research. Also, selected 23 Y-STR markers within PowerPlex Y 23 kit are very useful in the investigation of genetic relationships between examined populations.
